# Recent Developments in the Non-Pharmacological Management of Children’s Behavior Based on Distraction Techniques: A Concise Review

**DOI:** 10.3390/healthcare12191940

**Published:** 2024-09-27

**Authors:** Jieyi Chen, Ke Deng, Dikuan Yu, Cancan Fan, Limin Liu, Haijing Gu, Fang Huang, Yongbiao Huo

**Affiliations:** 1 Hospital of Stomatology, Sun Yat-sen University, Guangzhou 510055, China; chenjy679@mail.sysu.edu.cn (J.C.); yudk3@mail3.sysu.edu.cn (D.Y.); fancanc@mail.sysu.edu.cn (C.F.); liulmin@mail.sysu.edu.cn (L.L.); guhj@mail.sysu.edu.cn (H.G.); hfang@mail.sysu.edu.cn (F.H.); 2 Faculty of Dentistry, The University of Hong Kong, Hong Kong SAR, China; cdeng@hku.hk

**Keywords:** dental anxiety, oral health, virtual reality, behavior, child

## Abstract

Oral diseases and conditions affect children’s oral health and negatively influence their overall health. Early detection and intervention are important in mitigating these negative consequences. However, dental fear and anxiety (DFA) regarding dental procedures often hinder children from seeking necessary dental care. Non-pharmacological behavior management strategies, such as distraction techniques, are commonly adopted to manage children’s behaviors. Distraction techniques have been developed rapidly in recent years and are widely accepted by both health professionals and parents due to their noninvasive and low-cost nature. This concise review aims to summarize current distraction techniques applied during dental treatments, especially for children. The most commonly reported techniques for children are audio distraction, audio-visual distraction, tactile distraction, olfactory distraction, and gustatory distraction. Audio distraction techniques involving music and storytelling help children relax. Audio-visual distraction techniques help to divert children’s attention from the dental treatment. Tactile stimuli can reduce the transmission of pain signals. Olfactory stimuli can help children feel comfortable and relaxed. Gustatory distraction involving sweet substances can create a positive environment. These distraction techniques effectively reduce DFA in children and improve their satisfaction with dental procedures. As technology continues to develop, further research is needed to provide more robust, evidence-based guidance for dentists using distraction techniques.

## 1. Introduction

Oral diseases and conditions, such as dental caries and dental trauma, are prevalent among children worldwide. *Lancet* reported that untreated dental caries in deciduous teeth affect 9% of the child population, potentially leading to unremitting pain and infection [1]. The International Association of Dental Traumatology indicated that 25% of schoolchildren experience dental trauma that may need urgent or emergency dental care [2]. The development and progression of oral diseases and conditions can negatively impact children’s oral cavity, maxillofacial development, and overall psychological health [3]. Early detection and management of oral diseases and conditions are essential to mitigate these consequences. However, dental fear and anxiety (DFA) during dental procedures can lead to negative or aggressive behaviors and hinder children from seeking the necessary dental care [4]. The term DFA refers to intense negative emotions associated with dental procedures. DFA is not uncommon among children. A systematic review reported that DFA affects 37% of preschool children and 25.8% of schoolchildren worldwide. Preschool children with dental caries experience had a higher prevalence rate of DFA [5]. Given this challenge, non-pharmacological management techniques have emerged as crucial strategies for addressing DFA and helping children receive appropriate dental care.

Non-pharmacological management techniques have been suggested as an initial approach to managing children’s behavior during dental procedures. A recent qualitative review reported that children’s first dental visit shapes their attitude toward further treatments, and the application of behavioral management techniques is one of the factors affecting this experience [6]. In a conventional dental clinic setting, the unfamiliar environment and people may evoke anxiety in children, and their discomfort may be amplified during dental procedures, leading to resistant behavior. A survey revealed that children generally show lower levels of DFA in dental outreach programs conducted in a familiar setting, such as kindergartens [7]. An important distinction to be made is that children are not small adults. They undergo rapid physical and psychological development and form their understanding of the world through interactions with their environment. Their reactions to unfamiliar situations, such as fear or curiosity, can lead to avoidance or exploration, depending on their sense of security and comfort. Therefore, reducing DFA through non-pharmacological management techniques among children is essential to facilitating not only the process of dental treatment but also the psychological development of children. These techniques are less expensive, noninvasive, and more acceptable to parents and children than sedation or general anesthesia.

Various non-pharmacological management techniques, such as desensitization, tell-show-do, modeling, reinforcement, voice control, restraint, distraction, and cognitive behavioral therapy, have been developed and applied in dental practice [8]. These techniques are widely used and are suggested to be able to change children’s behavior effectively through long-term use. The representative non-pharmacological management technique is the tell-show-do technique. Through explaining, demonstrating, and performing the procedure, children can better understand what they will experience and may be more receptive to the subsequent procedure. Nowadays, different distraction techniques are emerging due to the development of technology. These distraction techniques provide comfortable sensory stimulation through auditory sense, visual sense, tactile sense, olfaction, and gustation to reduce children’s attention to specific threatening stimuli during dental treatments, consequently reducing DFA. Clinical evidence suggests that distraction techniques can effectively alleviate children’s DFA because external stimuli from different senses can easily overload children’s focus and divert their attention from the dental treatment [9]. Understanding the new developments in distraction techniques may help dental practitioners choose an appropriate strategy when handling young children and help promote research and manufacturing in this field. This concise review summarizes the current distraction techniques applied during dental treatments, especially with children.

## 2. Audio Distraction Techniques

Hearing is one of the most important channels through which humans receive external information. The huge array of sounds in the environment alerts humans, even making us avoid the origin of a sound involuntarily. In the meantime, our instinct focuses its attention on the sound to determine whether there is any danger. Studies have shown that extreme dental anxiety and moderate dental anxiety are associated with drilling, which has a high frequency and decibel noise [10]. Audio distraction may help divert children’s attention from the sound of drilling. Commonly reported audio distraction techniques include the use of music and storytelling.

### 2.1. Music

Music is a common way to relax. As a noninvasive relaxation method, it is widely accepted and used. There are two ways to employ music to relieve DFA clinically. One method is passive, simply playing music and allowing patients to listen to music they like. The other is music therapy, which requires patients to cooperate with the music therapist and participate actively.

#### 2.1.1. Passive Music Intervention

Music interventions have the potential as alternatives to sedatives or anti-anxiety drugs for reducing patients’ preoperative anxiety [11]. The Cochrane review reported that music intervention had a beneficial effect in reducing anxiety among patients with coronary heart disease. Moreover, allowing patients to listen to their preferred music may maximize the anxiety-reducing effects [12]. However, another study reported that subject-selected music and experimenter-chosen music made no difference in relieving tension and enhancing relaxation [13]. Some researchers have investigated the effectiveness of different types of music in reducing anxiety. It was reported that stimulative music can lead to higher anxiety scores but reduced anticipation and can interfere with concentration when compared to sedative music [14]. Furthermore, instrumental music was reported to be more effective in reducing DFA than nursery rhymes [15]. It has also been suggested that music may be used alongside other behavior management techniques to reduce DFA in children and can be applied to all patients [16].

#### 2.1.2. Active Music Therapy

Music therapy is the clinical use of music to accomplish individualized goals such as reducing anxiety, improving mood, and promoting self-expression. It requires a professional music therapist to guide patients in listening, singing, playing instruments, or composing music. It is more time-consuming compared to passive music interventions. It is personalized and designed according to each patient’s demands and aims to increase active participation from patients. Intervention measures may include (I) active focus on attention, (II) deep breathing with music guidance, (III) music auxiliary relaxation, and (IV) music guidance [17]. Before surgery, active music therapy can help children leave their parents and enter the operating room, although music therapy may not alleviate anxiety during the induction of anesthesia [18].

### 2.2. Storytelling

Children love stories. Research has shown that storytelling can effectively reduce preoperative nerves and DFA among children [19,20]. The scenario is similar to bedtime storytelling by parents, making children more relaxed mentally and physically. Even newborn babies can hear a wide variety of sounds. They can turn their eyes and heads in the direction of the sounds [21]. This is why we consider attention to sound a natural human ability. Therefore, whether using music or a story, the use of audio distraction techniques to divert a child’s attention could be considered to manage children’s behavior during dental procedures.

## 3. Audio-Visual Distraction Techniques

Audio-visual distraction techniques are other frequently used methods of distraction. The most common method is to play videos, including movies, TV series, and animations, to attract patients’ attention. Patients, especially children, may even enjoy watching these videos. Meanwhile, there are also video games in which children can actively participate. These audio-visual games require children to focus, distracting them from the dental treatment. Some studies show that the use of audio-visual distraction techniques during dental treatment can alleviate children’s dental anxiety, particularly those who need treatment under local anesthesia [22,23].

### 3.1. Passive Audio-Visual Distraction Techniques

Using passive audio-visual distraction techniques does not require children’s cognitive engagement or active participation. Children can passively receive information without interacting with the audio-visual source, making it difficult to determine the effect of distraction on children. The effectiveness of attention diversion is usually indirectly assessed by observing whether the children laugh or refrain from answering the dentists’ questions.

#### 3.1.1. Distraction via the Screen of Electronic Devices

The screens of various electronic devices can be used as carriers for audio-visual distraction as long as they can play videos; such devices include mobile phones, televisions, and tablets. Studies have shown that using tablets to watch animations during the recovery phase of venipuncture helps reduce children’s anxiety [24]. It is also reported that watching TV can more effectively manage children’s DFA than listening to music with headphones [25]. Furthermore, using a laptop to watch animations is more effective than the tell-show-do technique in reducing children’s DFA. The combination of watching animations and using tell-show-do is preferable.

#### 3.1.2. Distraction via Audio/Visual Eyeglasses

Audio/visual eyeglasses effectively relieve children’s anxiety and discomfort during dental procedures [26]. Researchers revealed that audio/visual eyeglasses not only reduced DFA during dental treatment among children but also improved positive feedback after local anesthesia [27]. Studies suggested that the application of audio/visual eyeglasses during local anesthesia was as effective in reducing DFA as traditional behavior management techniques such as the tell-show-do technique [28,29]. Moreover, using audio/visual glasses was suggested to be a useful option for patients with mild-to-moderate DFA [30].

#### 3.1.3. Virtual Reality Technology

Virtual reality technology is believed to be a better method of distraction for managing children’s anxiety compared to distraction via the screens of electronic devices [31]. It can reduce the fear of medical procedures among people with different kinds of phobias or anxieties [32]. Some studies have illustrated that virtual reality is an effective tool for improving children’s behavior and reducing their perception of pain during caries removal and restoration, especially in patients with mild-to-moderate DFA [33,34]. A study reported that the use of virtual reality distraction could be an effective intervention to reduce pain among children when undergoing needle-related procedures [35]. Compared with using tablets, virtual reality significantly reduced pain and anxiety among school-aged children during phlebotomy [36]. Virtual reality was more acceptable among children between the ages of 8 and 10, and they behaved better in future dental appointments after trying the virtual reality equipment [37]. Using 3D video glasses or music as distraction techniques during local analgesia was found to be beneficial in most children 7–10 years of age, with 3D video glasses showing greater efficacy than music. Children who received treatment with 3D video glasses reported high levels of satisfaction [38]. The application of immersive virtual reality can distract children’s focus and increase their enjoyment during dental procedures [39]. Moreover, virtual reality is more effective in relieving injection anxiety related to tooth extraction and pulpectomy than counter-stimulation [40]. In general, virtual reality seems to be more effective than screen devices, music listening, and counter-stimulation and affects children with mild-to-moderate fear and anxiety. Older children are more likely to enjoy and be satisfied with virtual reality. Nevertheless, it is imperative that all of these findings are substantiated by robust, evidence-based medical research.

### 3.2. Active Audio-Visual Distraction Techniques

Active audio-visual distraction techniques require active participation from patients. Compared to passive video viewing, video games require patients to concentrate, which may divert their attention. Tablet-based interactive distraction was reported to be more useful than oral midazolam in reducing preoperative and post-anesthesia anxiety [41]. Both interactive games and cartoons are effective in reducing anxiety among patients aged four to eight. Some studies have shown that mobile games can be more effective in reducing anxiety or rejection behaviors before surgery and tooth extraction compared with watching cartoons [42,43]. However, interestingly, both children and clinicians seemed to prefer cartoons during dental visits over interactive games [44].

Humans are visual-oriented and heavily rely on vision rather than other senses. Newborn babies have underdeveloped visual capabilities compared to other senses and have poor focus and perception of color. When infants reach the age of two months, they can focus on objects as well as adults can, and their color vision is as well developed as adults by the age of four months [21]. Children are easily attracted to cartoons as they are full of animations and colors. Different types of video equipment are emerging to distract children’s attention, and they are becoming lighter and thinner due to the development of modern science and technology.

## 4. Tactile Stimulation Distraction Techniques

According to the gate control theory of pain, pain signals are transmitted through nerve fibers that carry information from the site of injury to the spinal cord and then to the brain [45]. However, the transmission of these signals can be inhibited or modulated by other sensory inputs that are not related to pain and activate nerve fibers to interfere with the transmission of pain signals. The theory proposes the existence of a “gate” in the spinal cord that can either block or allow the transmission of pain signals to the brain. This gate is influenced by different factors, such as the intensity of the pain, the context of the pain, and the emotional state of the individual. This theory suggests that the gate can be closed by activating non-painful sensory inputs, such as touch or vibration, reducing the transmission of pain signals and providing relief from pain.

### 4.1. Passive Tactile Distraction Techniques

#### 4.1.1. Counter-Stimulation

Counter-stimulation is gentle stroking or vibration of the mucosa, using a finger while applying slight pressure adjacent to the site of intraoral injection. This technique has been found to alleviate pain during dental injections among children [46]. Not only oral vibrators but also external vibrations caused by equipment such as Buzzy devices can reduce the pain of dental injections [47,48]. For children undergoing pulp therapy or extraction under local anesthesia, these external vibration devices exhibit better effects in alleviating needle-associated anxiety compared to using stroking with fingers [49].

#### 4.1.2. Massage

Some dental practitioners use eye massager equipment to manage DFA during a local anesthetic. It was reported that such a strategy could help patients relax and complete the treatment [50]. However, one study demonstrated that local massage before injection did not affect injection pain [51]. Therefore, the effectiveness of massage in reducing DFA remains unclear.

### 4.2. Active Tactile Distraction Techniques

Active touch requires children’s active participation. During the process, no vibration device is used to provide tactile stimulation. Children are instructed to interact with their environment to actively experience tactile stimuli.

#### 4.2.1. Distraction via Stress Balls

A plethora of studies have reported that anxiety is reduced by using a stress ball [52]. Pain and anxiety relief was achieved by using a stress ball to distract children’s attention during phlebotomy [53]. Nevertheless, distraction via a stress ball or audio-visual eyeglasses during local anesthesia was reported to have an influence on reducing DFA but did not significantly improve children’s anxiety or pain levels or their behavior when compared to other behavior management strategies [28].

#### 4.2.2. Animal-Assisted Therapy

Pet-assisted anti-anxiety therapy was reported to be a safe, effective, and inexpensive approach to reducing DFA and has profound potential to improve oral health [54]. It is suggested that animal-assisted therapy is an effective behavior management strategy for children aged 5–10 [55]. Current evidence shows that animal-assisted therapy can relieve children’s pain to some extent [56]. It could be a promising method of anxiety control for children, and high levels of satisfaction were observed among pediatric animal-assisted therapy users [57,58].

Moreover, animal-assisted therapy in an outpatient setting can significantly reduce pain and emotional distress for chronic pain patients [59]. For example, during dental procedures, patients demonstrated a reduction in anxiety in the accompany of a therapy dog and improved their satisfaction with the overall experience [60]. Numerous studies show that parents and children prefer dogs as therapy animals and have a positive attitude toward animal-assisted therapy [61]. Most people acknowledge that therapy animals in the clinic make them feel happy and less anxious [62]. Therefore, a comfortable touch can also relax patients and distract their attention. Nevertheless, further research should be conducted to confirm the effectiveness of active or passive tactile distraction before and during dental treatment.

## 5. Olfactory Stimulation Distraction Techniques

Fragrance can make people feel certain emotions. For example, lavender oil is commonly used as it positively reduces anxiety among children when used appropriately [63]. The use of lavender aromas in dental settings was reported to reduce children’s DFA and also to reduce pain perception during local anesthetic injection [64]. As a result, lavender oil was suggested for daily practice in a pediatric dental setting [65]. One study showed that lavender oil inhalation was more effective in reducing children’s DFA when compared with audio-visual distraction during tooth extraction [66].

Another example is using aromatherapy with natural essential oils extracted from oranges. Orange essential oils were reported to help reduce salivary cortisol levels and pulse rates during dental treatment, particularly among 6- to 9-year-old children at their first dental visit [67,68]. Further investigation showed that lavender and orange essential oils effectively manage DFA among 6- to 12-year-old children when administered using a nebulizer or inhaler [69,70].

Aromatherapy can effectively alleviate children’s dental anxiety [71,72]. Furthermore, a combination of music therapy and aromatherapy was suggested to be more effective than single therapy in reducing children’s DFA [73].

## 6. Gustatory Stimulation Distraction Techniques

Taste distraction techniques using lollipops provide a positive environment and reduce DFA in children during intraoral periapical radiography [74]. It has been reported that if children were given a sweet taste before dental injections, their pain was alleviated. The influence of this effect is determined by the individual’s taste for sweetness and their body mass index [75]. Another study recommends honey as a sweet taste because it can reduce the pain caused by injections and promote children’s cooperation [76]. Newborns can distinguish several basic tastes. Like adults, they relax their facial muscles in response to sweetness, purse their lips when the taste is sour, and show archlike mouth openings when it is bitter [21]. Based on human nature, sweets have the potential to be used to manage children’s behavior during dental treatment.

## 7. Discussion

This concise review summarized the updated distraction techniques and their potential to reduce DFA among children in dental practice. Nowadays, distraction techniques are commonly used to manage DFA among children. These techniques appear to be effective in reducing mild-to-moderate DFA in children. However, they may not be as effective for those with extreme dental anxiety. In such cases, sedatives like midazolam or general anesthesia may be necessary to assist uncooperative children. However, parents often express concerns about the potential harm or adverse effects on their children’s mental development, making pharmacological management a last resort for them [77]. In contrast, non-pharmacological distraction techniques are generally accepted by dental practitioners, parents, and children. These cost-effective and noninvasive methods are worth trying among dental practitioners during children’s dental visits.

Distraction techniques, especially those that stimulate the auditory or visual senses, have progressed rapidly in recent years, along with the development of technology. Multimedia digital devices are becoming smaller and lighter, but their screens are larger, and their functions are more diverse. Children can have a better experience watching videos or playing games on these devices. Furthermore, the videos or games are varied, inexhaustible, and designed to cater to children’s tastes. Children are easily attracted to these videos or games and spend an increasing amount of time using digital devices [78]. Under such circumstances, audio and visual distraction techniques are promoted and developed. Moreover, clinicians are constantly exploring and introducing new equipment and techniques to help facilitate dental treatment procedures and provide a better experience for their patients by promoting the development of distraction techniques. Some pediatric dentists even send digitalized pre-visit imagery to children through WhatsApp as a time-saving approach to reduce their DFA. Such pre-visit watching or reading materials were shown to be an effective alternative to managing children’s DFA [79]. Furthermore, employing multiple distraction techniques yields greater effectiveness concurrently. The diverse environmental stimuli are designed to relax children and foster cooperative engagement during treatment procedures, which aligns with the objective of managing children’s behavior effectively. These distraction techniques are booming and attracting many researchers and manufacturers’ interest.

These new distraction techniques not only benefit the average child but also have a positive effect on children with special needs. Researchers found that music can reduce DFA among children with an intellectual disability [80]. Audio and visual reality distraction techniques were reported to successfully reduce DFA in children with mild intellectual disability or speech and hearing disabilities [81,82]. Though these distraction techniques are widely used nowadays, caution should be taken when choosing appropriate children-friendly content, and guidelines should be formulated to reduce the risk of indulgence in digital devices.

## 8. Conclusions

In conclusion, distraction techniques can help reduce dental fear and anxiety in children and improve their satisfaction with dental procedures. Applying multiple distraction methods together may yield superior outcomes compared to non-pharmacological management approaches. As equipment and techniques continue to develop, further research is needed to provide more robust, evidence-based guidance for dentists using these distraction techniques.

## Data Availability

No new data were created or analyzed in this study. Data sharing is not applicable to this article.

## References

[1] Peres M.A., Macpherson L.M., Weyant R.J., Daly B., Venturelli R., Mathur M.R., Listl S., Celeste R.K., Guarnizo-Herreño C.C., Kearns C. (2019). Oral diseases: A global public health challenge. Lancet.

[2] Levin L., Day P.F., Hicks L., O’Connell A., Fouad A.F., Bourguignon C., Abbott P.V. (2020). International Association of Dental Traumatology guidelines for the management of traumatic dental injuries: General introduction. Dent. Traumatol..

[3] Gomes M.C., Pinto-Sarmento T.C.d.A., Costa E.M.M.d.B., Martins C.C., Granville-Garcia A.F., Paiva S.M. (2014). Impact of oral health conditions on the quality of life of preschool children and their families: A cross-sectional study. Health Qual. Life Outcomes.

[4] Milgrom P., Newton J.T., Boyle C., Heaton L.J., Donaldson N. (2010). The effects of dental anxiety and irregular attendance on referral for dental treatment under sedation within the National Health Service in London. Community Dent. Oral Epidemiol..

[5] Grisolia B.M., Dos Santos A.P.P., Dhyppolito I.M., Buchanan H., Hill K., Oliveira B.H. (2021). Prevalence of dental anxiety in children and adolescents globally: A systematic review with meta-analyses. Int. J. Paediatr. Dent..

[6] Tahani B., Nilchian F. (2024). Children’s experiences during their first dental visit: A qualitative study. Community Dent. Health.

[7] Yon M.J.Y., Chen K.J., Gao S.S., Duangthip D., Lo E.C.M., Chu C.H. (2020). Dental fear and anxiety of kindergarten children in Hong Kong: A cross-sectional study. Int. J. Environ. Res. Public Health.

[8] Roberts J., Curzon M., Koch G., Martens L. (2010). Behaviour management techniques in paediatric dentistry. Eur. Arch. Paediatr. Dent..

[9] Prado I.M., Carcavalli L., Abreu L.G., Serra-Negra J.M., Paiva S.M., Martins C.C. (2019). Use of distraction techniques for the management of anxiety and fear in paediatric dental practice: A systematic review of randomized controlled trials. Int. J. Paediatr. Dent..

[10] Moore R., Birn H., Kirkegaard E., Brødsgaard I., Scheutz F. (1993). Prevalence and characteristics of dental anxiety in Danish adults. Community Dent. Oral Epidemiol..

[11] Bradt J., Dileo C., Shim M. (2013). Music interventions for preoperative anxiety. Cochrane Database Syst. Rev..

[12] Bradt J., Dileo C., Potvin N. (2013). Music for stress and anxiety reduction in coronary heart disease patients. Cochrane Database Syst. Rev..

[13] Thaut M.H., Davis W.B. (1993). The influence of subject-selected versus experimenter-chosen music on affect, anxiety, and relaxation. J. Music. Ther..

[14] Smith C.A., Morris L.W. (1977). Differential effects of stimulative and sedative music on anxiety, concentration, and performance. Psychol. Rep..

[15] Marwah N., Prabhakar A., Raju O. (2005). Music distraction-its efficacy in management of anxious pediatric dental patients. J. Indian Soc. Pedod. Prev. Dent..

[16] Ainscough S., Windsor L., Tahmassebi J. (2019). A review of the effect of music on dental anxiety in children. Eur. Arch. Paediatr. Dent..

[17] Bradt J., Teague A. (2018). Music interventions for dental anxiety. Oral Dis..

[18] Kain Z.N., Caldwell-Andrews A.A., Krivutza D.M., Weinberg M.E., Gaal D., Wang S.-M., Mayes L.C. (2004). Interactive music therapy as a treatment for preoperative anxiety in children: A randomized controlled trial. Anesth. Analg..

[19] Amer H.W., Mohamed H.M.M.F., Ali S.A.O., Souilm N.A.M., Zaghamir D.E.F. (2021). Effect of Storytelling on Preoperative Anxiety and Fear among Children Undergoing Surgery. Egypt. J. Health Care.

[20] Castronuevo L.A., Arguelles K.G., Rinkima R., Orbon M., Subido H., Orais R., Gumarao M., Taclan L. (2018). Effectiveness of story telling on children’s dental anxiety. J. Health Sci..

[21] Berk L.E. (2022). Development through the Lifespan.

[22] Liu Y., Gu Z., Wang Y., Wu Q., Chen V., Xu X., Zhou X. (2019). Effect of audiovisual distraction on the management of dental anxiety in children: A systematic review. Int. J. Paediatr. Dent..

[23] Zhang C., Qin D., Shen L., Ji P., Wang J. (2019). Does audiovisual distraction reduce dental anxiety in children under local anesthesia? A systematic review and meta-analysis. Oral Dis..

[24] Lee H.N., Hwang S., Jung J.Y., Park J.W., Kim D.K., Kwak Y.H. (2022). Tablet personal computer distraction during intravenous placement for young children in the pediatric emergency department: A pilot study. Pediatr. Int..

[25] Prabhakar A., Marwah N., Raju O. (2007). A comparison between audio and audiovisual distraction techniques in managing anxious pediatric dental patients. J. Indian Soc. Pedod. Prev. Dent..

[26] Ram D., Shapira J., Holan G., Magora F., Cohen S., Davidovich E. (2010). Audiovisual video eyeglass distraction during dental treatment in children. Quintessence Int..

[27] Al-Khotani A., Bello L.A., Christidis N. (2016). Effects of audiovisual distraction on children’s behaviour during dental treatment: A randomized controlled clinical trial. Acta Odontol. Scand..

[28] Shekhar S., Suprabha B., Shenoy R., Rao A., Rao A. (2022). Effect of active and passive distraction techniques while administering local anaesthesia on the dental anxiety, behaviour and pain levels of children: A randomised controlled trial. Eur. Arch. Paediatr. Dent..

[29] Shah U., Bhatia R. (2018). Effectiveness of audiovisual distraction eyeglass method compared to tell-play-do technique among 4–7-year-old children: A randomized controlled trial. Int. J. Oral Care Res..

[30] Frere C.L., Crout R., Yorty J., Mcneil D.W. (2001). Effects of audiovisual distraction during dental prophylaxis. J. Am. Dent. Assoc..

[31] Tailor B., Bargale S., Dave B.H., Deshpande A.N., Thomas P.S., Jain A. (2021). Comparison of virtual reality glasses vs on-screen distraction technique in reduction of pediatric dental anxiety: An in vivo study. J. South Asian Assoc. Pediatr. Dent..

[32] Kılıç A., Brown A., Aras I., Hui R., Hare J., Hughes L.D., McCracken L.M. (2021). Using virtual technology for fear of medical procedures: A systematic review of the effectiveness of virtual reality-based interventions. Ann. Behav. Med..

[33] Custódio N.B., Costa F.d.S., Cademartori M.G., da Costa V.P.P., Goettems M.L. (2020). Effectiveness of virtual reality glasses as a distraction for children during dental care. Pediatr. Dent..

[34] Wiederhold M.D., Gao K., Wiederhold B.K. (2014). Clinical use of virtual reality distraction system to reduce anxiety and pain in dental procedures. Cyberpsychol. Behav. Soc. Netw..

[35] Czech O., Wrzeciono A., Rutkowska A., Guzik A., Kiper P., Rutkowski S. (2021). Virtual reality interventions for needle-related procedural pain, fear and anxiety—A systematic review and meta-analysis. J. Clin. Med..

[36] İnangil D., Şendir M., Büyükyılmaz F. (2020). Efficacy of cartoon viewing devices during phlebotomy in children: A randomized controlled trial. J. Perianesth. Nurs..

[37] Al-Halabi M.N., Bshara N., AlNerabieah Z. (2018). Effectiveness of audio visual distraction using virtual reality eyeglasses versus tablet device in child behavioral management during inferior alveolar nerve block. Anaesth. Pain. Intensive Care.

[38] Nuvvula S., Alahari S., Kamatham R., Challa R. (2015). Effect of audiovisual distraction with 3D video glasses on dental anxiety of children experiencing administration of local analgesia: A randomised clinical trial. Eur. Arch. Paediatr. Dent..

[39] Atzori B., Lauro Grotto R., Giugni A., Calabrò M., Alhalabi W., Hoffman H.G. (2018). Virtual reality analgesia for pediatric dental patients. Front. Psychol..

[40] Nunna M., Dasaraju R.K., Kamatham R., Mallineni S.K., Nuvvula S. (2019). Comparative evaluation of virtual reality distraction and counter-stimulation on dental anxiety and pain perception in children. J. Dent. Anesth. Pain. Med..

[41] Stewart B., Cazzell M.A., Pearcy T. (2019). Single-blinded randomized controlled study on use of interactive distraction versus oral midazolam to reduce pediatric preoperative anxiety, emergence delirium, and postanesthesia length of stay. J. Perianesth. Nurs..

[42] Allani S., Setty J.V. (2016). Effectiveness of distraction techniques in the management of anxious children in the dental operatory. IOSR J. Dent. Med. Sci..

[43] Kumprasert P., Prapansilp W., Rirattanapong P. (2021). Video games, audiovisual, and conventional distractions for pediatric dental patients: A crossover randomized controlled clinical trial. Mahidol Dent. J..

[44] Venkiteswaran A., Halim R.A., Hasmun N.N. (2018). Effectiveness of Two Audiovisual Distractions for Paediatric Patients Undergoing Restorative Treatment. J. Multidiscip. Res..

[45] Moayedi M., Davis K.D. (2013). Theories of pain: From specificity to gate control. J. Neurophysiol..

[46] Aminabadi N.A., Farahani R.M.Z., Balayi Gajan E. (2008). The efficacy of distraction and counterstimulation in the reduction of pain reaction to intraoral injection by pediatric patients. J. Contemp. Dent. Pract..

[47] Hegde K.M., Srinivasan I., DR M.K., Melwani A., Radhakrishna S. (2019). Effect of vibration during local anesthesia administration on pain, anxiety, and behavior of pediatric patients aged 6–11 years: A crossover split-mouth study. J. Dent. Anesth. Pain. Med..

[48] Ching D., Finkelman M., Loo C.Y. (2014). Effect of the DentalVibe injection system on pain during local anesthesia injections in adolescent patients. Pediatr. Dent..

[49] Sahithi V., Saikiran K.V., Nunna M., Elicherla S.R., Challa R.R., Nuvvula S. (2021). Comparative evaluation of efficacy of external vibrating device and counterstimulation on child’s dental anxiety and pain perception during local anesthetic administration: A clinical trial. J. Dent. Anesth. Pain. Med..

[50] Kunusoth R., Colvenkar S., Alwala A.M., Sampreethi S., Ahmed M.S. (2022). Massage therapy to control anxiety before extraction of an impacted tooth. Cureus.

[51] Sharifi R., Khazaei S., Mozaffari H.R., Amiri S.M., Iranmanesh P., Mousavi S.A. (2017). Effect of massage on the success of anesthesia and infiltration injection pain in maxillary central incisors: Double-blind, crossover trial. Dent. Hypotheses.

[52] Linthoingambi A., Harsimran K., Rishika C., Ramakrishna Y. (2022). Effectiveness of intellectual color game, audio-visual and stress ball distraction methods on gagging and anxiety management in children. J. Clin. Pediatr. Dent..

[53] Aydin D., Şahiner N.C., Çiftçi E.K. (2016). Comparison of the effectiveness of three different methods in decreasing pain during venipuncture in children: Ball squeezing, balloon inflating and distraction cards. J. Clin. Nurs..

[54] Manley L. (2016). On the use of pets to manage dental anxiety. Dent. Hypotheses.

[55] Thakkar T., Naik S., Dixit U. (2021). Assessment of dental anxiety in children between 5 and 10 years of age in the presence of a therapy dog: A randomized controlled clinical study. Eur. Arch. Paediatr. Dent..

[56] Zhang Y., Yan F., Li S., Wang Y., Ma Y. (2021). Effectiveness of animal-assisted therapy on pain in children: A systematic review and meta-analysis. Int. J. Nurs. Sci..

[57] Nammalwar R.B., Rangeeth P. (2018). A bite out of anxiety: Evaluation of animal-assisted activity on anxiety in children attending a pediatric dental outpatient unit. J. Indian Soc. Pedod. Prev. Dent..

[58] Charowski M., Wells M.H., Dormois L., Fernandez J.A., Scarbecz M., Maclin M. (2021). A randomized controlled pilot study examining effects of animal assisted therapy in children undergoing sealant placement. Pediatr. Dent..

[59] Marcus D.A., Bernstein C.D., Constantin J.M., Kunkel F.A., Breuer P., Hanlon R.B. (2012). Animal-assisted therapy at an outpatient pain management clinic. Pain Med..

[60] Cruz-Fierro N., Vanegas-Farfano M., González-Ramírez M.T. (2019). Dog-assisted therapy and dental anxiety: A pilot study. Animals.

[61] Gupta N., Yadav T. (2018). Parents’ acceptance and their children’s choice of pet for animal-assisted therapy (AAT) in 3-to 12-year-old children in the dental operatory–A questionnaire-based pilot study. Int. J. Paediatr. Dent..

[62] Cass K.D. (2021). Animal Assisted Therapy: A Survey of Patient’s and Caregivers’ Perceptions in Orthodontics. Master’s Thesis.

[63] Gandhi H., Panda A., Kaur M., Natasha N., Kotecha V., Shah S. (2016). Effect of lavender oil on anxiety level in pediatric patients. Int. J. Curr. Res..

[64] Ghaderi F., Solhjou N. (2020). The effects of lavender aromatherapy on stress and pain perception in children during dental treatment: A randomized clinical trial. Complement. Ther. Clin. Pract..

[65] Arslan I., Aydinoglu S., Karan N.B. (2020). Can lavender oil inhalation help to overcome dental anxiety and pain in children? A randomized clinical trial. Eur. J. Pediatr..

[66] Mohamed Khattab A., Mahmoud Abd Elhamid Dawood B., Mohammed Ahmed Gado E., Mohammed Elsayed Sharshor S. (2022). Effect of Aromatherapy versus Audiovisual Distraction on pain and anxiety of children undergoing Dental Extraction. Int. Egypt. J. Nurs. Sci. Res..

[67] Jafarzadeh M., Arman S., Pour F.F. (2013). Effect of aromatherapy with orange essential oil on salivary cortisol and pulse rate in children during dental treatment: A randomized controlled clinical trial. Adv. Biomed. Res..

[68] Soni S., Bhatia R., Oberoi J. (2018). Evaluation of the efficacy of aromatherapy on anxiety level among pediatric patients in a dental setting: A randomized control trial. Int. J. Oral Care Res..

[69] Abdalkhalek W. (2023). Clinical Evaluation of The Effect of volatile oils on Anxiety of Children During Dental procedures: An in-Vivo Study. Egypt. Dent. J..

[70] Nirmala K., Kamatham R. (2021). Effect of aromatherapy on dental anxiety and pain in children undergoing local anesthetic administrations: A randomized clinical trial. J. Caring Sci..

[71] Pasupuleti S.C., Hassan A. (2022). Evaluation of effectiveness of aromatherapy in managing anxious paediatric dental patient: An in-vivo study. J. MAR Dent. Sci..

[72] Purohit A., Singh A., Purohit B., Shakti P., Shah N. (2021). Is aromatherapy associated with patient’s dental anxiety levels? A systematic review and meta-analysis. J. Dent. Anesth. Pain Med..

[73] Janthasila N., Keeratisiroj O. (2023). Music therapy and aromatherapy on dental anxiety and fear: A randomized controlled trial. J. Dent. Sci..

[74] Tyagi P., Mali S., Rathi S.V., Agrawal N., Kumar A., Abraham J.M. (2022). Comparative evaluation of visual and taste distraction techniques using rms pictorial scale in making of periapical radiographs. J. South Asian Assoc. Pediatr. Dent..

[75] Ghaderi F., Ahmadbeigi M., Vossoughi M., Sardarian A. (2021). The efficacy of administrating a sweet-tasting solution for reducing the pain related to dental injections in children: A randomized controlled trial. Int. J. Paediatr. Dent..

[76] Janiani P., Gurunathan D. (2021). Effectiveness of pre-administered natural sweet-tasting solution for decreasing pain associated with dental injections in children: A split-mouth randomized controlled trial. J. Contemp. Dent. Pract..

[77] McCann M.E., Soriano S.G. (2019). Does general anesthesia affect neurodevelopment in infants and children?. BMJ.

[78] Nathan T., Muthupalaniappen L., Muhammad N.A. (2022). Prevalence and description of digital device use among preschool children: A cross-sectional study in Kota Setar District, Kedah. Malays. Fam. Physician.

[79] Reddy R.E., Merum K., Mudusu S.P., Srikanth S., Dubey P. (2021). Effect of Digitalized Previsit Imagery on Behavior of Children in the Dental Operatory. Int. J. Clin. Pediatr. Dent..

[80] Gowdham G., Shetty A.A., Hegde A., Suresh L.R. (2021). Impact of Music Distraction on Dental Anxiety in Children Having Intellectual Disability. Int. J. Clin. Pediatr. Dent..

[81] Mehrotra D., Manju R. (2023). Comparative evaluation of the effect of audio and virtual reality distraction on the dental anxiety of healthy and mild intellectually disabled children. J. Indian Soc. Pedod. Prev. Dent..

[82] Kaur J., Shivashankarappa P.G., Sanguida A., Suganya M., Ezhumalai G. (2021). Effectiveness of Visual Distraction with and without Virtual Reality Glasses in Reducing Dental Anxiety among Children with Hearing and Speech Disability: A Pilot Study. Int. J. Clin. Pediatr. Dent..

